# Barriers and facilitating factors in the prevention of diabetes type 2 and gestational diabetes in vulnerable groups: A scoping review

**DOI:** 10.1371/journal.pone.0232250

**Published:** 2020-05-13

**Authors:** Jessica Breuing, Dawid Pieper, Annika Lena Neuhaus, Simone Heß, Lena Lütkemeier, Fabiola Haas, Mark Spiller, Christine Graf

**Affiliations:** 1 Department for Evidence Based Health Services Research, Department of Medicine, Faculty of Health, Institute for Research in Operative Medicine (IFOM), Witten / Herdecke University, Cologne, Germany; 2 Institute of Movement and Neurosciences, German Sport University Cologne, Cologne, Germany; University Lyon 1 Faculty of Dental Medicine, FRANCE

## Abstract

**Aims:**

Type 2 diabetes mellitus (T2DM) and gestational diabetes (GDM) are globally on the rise, accompanied by comorbidities and associated health costs. Increased physical activity, healthy nutrition, and weight loss have shown the potential to prevent T2DM/GDM. Despite this, reaching vulnerable groups remains a key challenge. The aim of this scoping review was to identify barriers and facilitating factors in the prevention of T2DM/GDM in vulnerable groups.

**Methods:**

We conducted a systematic literature search in May 2018, updated in September 2019, in several databases (e.g. PubMed, Embase) to identify barriers and facilitating factors in the prevention of T2DM/GDM in vulnerable groups. Two reviewers independently screened the results. Extracted data was charted, categorized, and summarized.

**Results:**

We included 125 articles. Ninety-eight studies were extracted, and eight categories of barriers and facilitating factors were formed. The most common categories of barriers were limited knowledge, family/friends, and economic factors, and the most common categories of facilitating factors were family/friends, social support, and knowledge.

**Conclusion:**

This scoping review identified various barriers and facilitating factors in vulnerable groups. Preventive interventions should consider these barriers and facilitating factors in developing preventive interventions or in adapting existing ones.

## Background

The prevalence of type 2 diabetes mellitus (T2DM) and gestational diabetes (GDM) is rising worldwide, and so are the associated health consequences and healthcare costs [1]. Evidence shows that the prevalence of T2DM/GDM is higher in obese or overweight, physically inactive individuals [2, 3]. Increased physical activity, healthy nutrition, and weight loss may prevent or delay T2DM/GDM manifestation [4]. Lifestyle interventions like the Diabetes Prevention Program (DDP) may reduce the risk of T2DM more effectively than antidiabetic drugs such as metformin [5]. However, these preventive interventions typically target patients mostlyfrom the general population, and it is challenging to reach vulnerable groups, including individuals with a migration background and/or low socio-economic status. Such patients are disproportionally affected by T2DM/GDM and diabetes-related complications [6, 7]. Language, cultural perception, and lower health literacy often play important roles in non-participation [8]. Research suggests that behavioral change is possible, but generally requires comprehensive approaches tailored to specific settings and target groups [9]. Therefore, the development of new T2DM/GDM interventions should be informed by evidence of barriers and facilitating factors. This may enhance the willingness of patients to participate in preventive interventions. The aim of this scoping review was to identify and describe barriers and facilitating factors in the prevention of T2DM/GDM in vulnerable groups.

## Methods

This project was commissioned by the Federal Centre for Health Education in Germany as part of the “National education and communication strategy on diabetes mellitus”. Its protocol was published a-priori [10]. Since the International prospective register of systematic reviews (PROSPERO) does not register scoping reviews, this scoping review is not registered.

The scoping review was conducted following the Arksey and O´Malleys framework [11] and the Joanna Briggs Institute Reviewers’ Manual 2015 [12]. It is reported based on the Preferred Reporting Items for Systematic reviews and Meta-Analyses extension for Scoping Reviews (PRISMA-ScR) Checklist [13].

### Eligibility criteria

Inclusion criteria

- vulnerable patients with, or at risk of, T2DM/GDM
- studies present barriers and facilitating factors for implementing a preventive intervention
- WHO mortality stratum A countries
- publication date ≥ 2008

Exclusion criteria

- indigenous people, children, or people with mental disorders
- no full texts available
- general prevention without any context of T2DM/GDM
- patients on antidiabetic medication

Eligibility criteria were categorized using the Population, Concept, Context (PCC) mnemonic (Table 1). All study types were eligible that present barriers to and facilitating factors for implementing preventive interventions in vulnerable patients with, or at risk of, T2DM/GDM. Studies published before January 2008 were excluded, because barriers and facilitators are affected by external factors such as accessibility of care and information. We assume that accessibility has changed substantially compared to 10 years ago due to the increased volume of digital and virtual goods, services, and processes in healthcare. As a result, the barriers and facilitators might have changed, so that there would be a lack of comparability if we chose a longer period.

**Table 1:** PCC (Population, Concept, Context).

P	Diabetes mellitus type 2 or gestation diabetes	Type 2 diabetes mellitus
		Gestational diabetes mellitus
		People at risk of developing diabetes mellitus or gestational diabetes mellitus
	Vulnerable patients/-groups	Elderly, older people, seniors > 65 years
		Disabled people
		People in need of care, residents of a nursing home
		Unemployed people
		Refugees/migrants as well as ethnic groups (e.g. African Americans or Hispanics)
		Homeless people
		Drug/substance abusers (excluding nicotine abuse/smoking)
		Low socio-economic status
C	Prevention	Primary/ secondary/ tertiary prevention
	Barriers and facilitating factors	Definition of barriers and motivating aspects e.g. language, costs, religion, ethnic background, low income, social and health support
		Solutions to exploit barriers and support solutions e.g. materials and manpower, use of media, insurance
C	>2008; WHO stratum A	
Other	all types of studies; all languages; available in full text version	

The PPC (Population, Concept, Context) mnemonic illustrates the eligibility criteria for the scoping review. Additionally to the classic PPC mnemonic there are other criteria regarding study types, languages and the availability of the full text version.

No language restriction was applied. All full texts published in languages other than English or German were translated by an external agency. We only included studies performed in countries within the low mortality stratum (A) defined by the World Health Organization [14]. By doing so, we ensured that our findings were applicable throughout western industrialized countries. We define vulnerable groups according to Lewis et al. [15], but excluded indigenous people, children, and people with mental disorders. This was done to align our study with the aims of the “National education and communication strategy on diabetes mellitus”. To separate tertiary prevention from therapy, we excluded studies with patients treated with any antidiabetic medication. Furthermore, we excluded studies which could be interpreted as preventing general metabolic risk factors without primary focus on T2DM/GDM prevention, such as studies aiming at weight reduction in obese patients.

### Information sources

The following electronic databases were searched: PubMed, EMBASE, PsycINFO, PSYNDEX, Social Science Citation Index, and CINAHL. Grey literature was searched on greylit.org and via the homepages of the WHO and international healthcare or public health departments (e.g. Department of Health & Social Care, UK; Agency for Healthcare Research and Quality (AHRQ); US Preventive Services Task Force). We manually checked the reference lists of all included studies.

### Search

The search strategy was developed by the research team in collaboration with an experienced librarian and checked by a referee according to the Peer Review of Electronic Search Strategies (PRESS) guideline [16]. All initial database searches were conducted in May 2018, while grey literature was searched in July 2018. The initial database searches were updated in September 2019. The initial grey literature search was not updated because the original search was very time-consuming and did not yield any relevant references. The search strategy is presented as S1 Appendix.

### Data management

The search results were uploaded and managed using Microsoft Excel.

### Study selection

Two reviewers independently screened the titles and abstracts of all search results and assessed full texts of potentially relevant articles against the predefined selection criteria. Any disagreement was resolved by discussion and consensus. The reasons for exclusion of full texts were documented.

### Data extraction

A standardized extraction form was developed for this review. Using a sample of five articles, the form was piloted, assessed for completeness and applicability, and modified to ensure all data necessary to address the research questions were obtained. Data were extracted by one reviewer and checked by another. Disagreements were resolved through discussion and consensus.

### Data items

The preliminary data extraction categories were derived from our overarching research question. The following data were collected:

- study characteristics (e.g. country, setting, publication date, number of participants, study design/method)
- patient characteristics (e.g. age, gender, allocation to vulnerable group)
- inclusion/exclusion criteria
- barriers
- facilitating factors

Patient allocation to a vulnerable group was made according to the PCC mnemonic. Studies targeting mixed groups were allocated to the new ‘mixed vulnerable group’. We assigned each barrier and facilitating factor to one of the following eight predefined categories: language, economic factors, family and friends, work, social support, religion, culture, and knowledge. We defined these categories based on qualitative thematic analysis. The total number of barriers and facilitating factors within each category is based on the number of identified studies which mentioned this barrier or facilitating factor. We did not analyze how often a barrier or facilitating factor is mentioned within each study.

### Risk of bias

As this is a scoping review, risk of bias was not assessed. This is consistent with guidance on the conduct of scoping reviews [11].

### Data analysis

We used Arksey and O’Malley’s methods [11] and provide a descriptive analysis of the extent, nature, and distribution of the studies included in the review as well as a narrative thematic summary of the data collected. This was achieved by summarizing the literature according to the types of vulnerable groups, comparators, barriers and prohibitive factors, and outcomes identified. We aimed to map the extent, range, and nature of research in this area using visual representations of the data. Data were charted, categorized, and summarized. We reported quantitative (e.g. frequency) and qualitative results. Furthermore, we sought to explore similarities and differences, both within and between studies, to identify patterns and themes and postulate explanations for findings. We focused on barriers and prohibitive factors for preventive interventions in vulnerable patients with or at risk of T2DM/GDM. We also considered the robustness of the included studies by assessing the overall strength and confidence of the findings. Where possible, we stratified our results by vulnerable groups.

## Results

**Fig 1:**
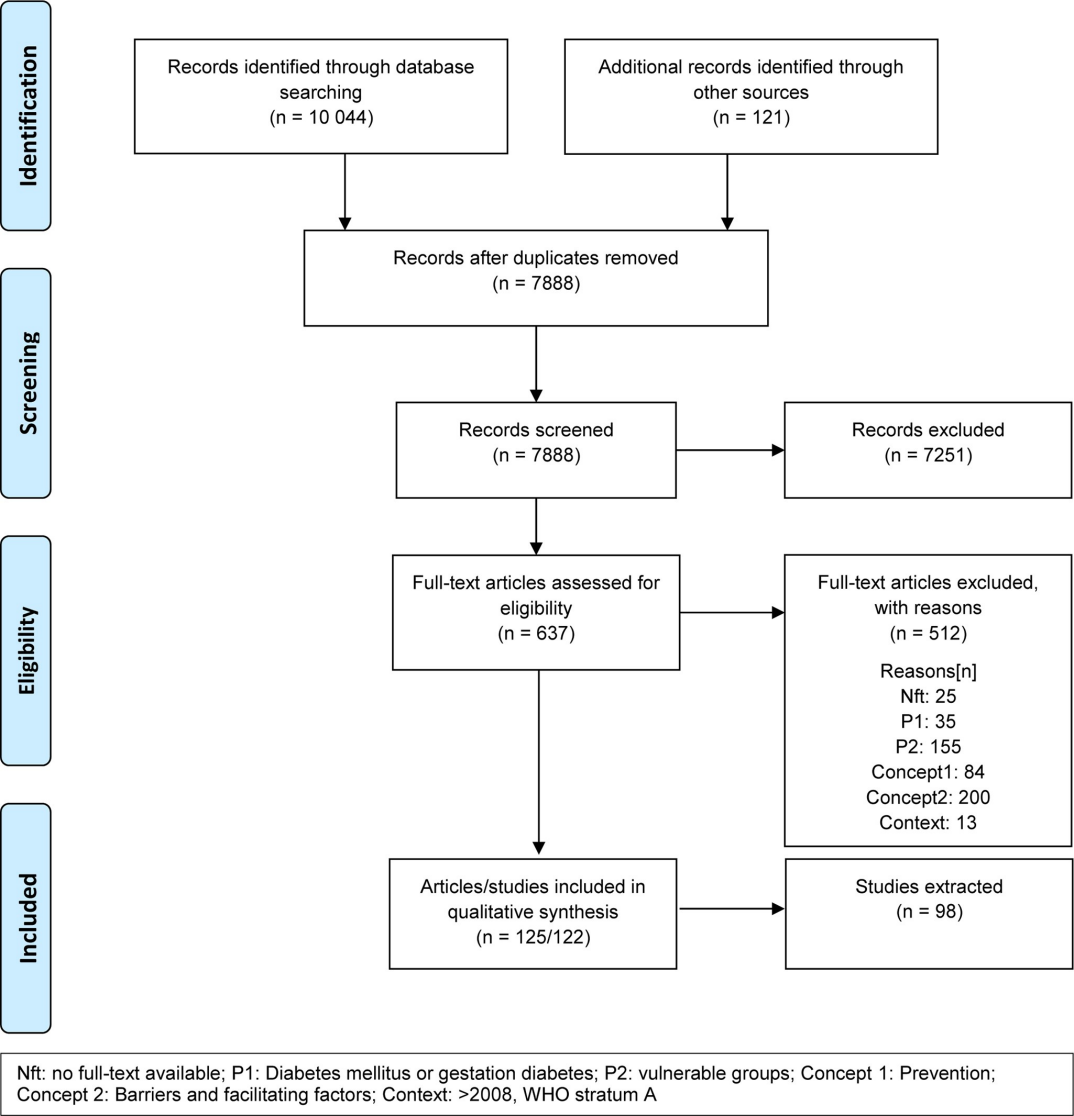
Flow chart.

Our database search yielded 10,044 articles. An additional 121 references were identified by searching for grey literature and checking reference lists, which led to 7888 articles after deduplication (see Fig 1). A list of excluded full-text articles is available as S2 Appendix. A total of 125 articles (122 studies) were included. Included reviews (n = 24, e.g. systematic reviews, narrative reviews) were used for reference-checking but no data were extracted. The remaining 101 articles (98 studies) were extracted and analyzed.

### Characteristics of included studies

Among included studies, n = 77 had a qualitative design (focus groups n = 40, interviews n = 37). Most studies were conducted in the US (n = 65), others in Australia (n = 8), Canada (n = 7), and the UK (n = 8). We identified four studies which focused only on GDM [17–20], and three (four articles) which focused on both T2DM and GDM [21–24]. The most prominent vulnerable groups within these studies were migrants (n = 59), ethnic groups (n = 23) such as Afro-Americans, and people with low socio-economic status (n = 6). Other vulnerable groups identified were older migrants (n = 2), migrants/ethnic groups which could not be sharply delineated (n = 2), homeless people (n = 1), a mixed group of people with a low socioeconomic status and homeless people (n = 1), unemployed people (n = 1), migrants with low socio-economic status (n = 1), people with disabilities (n = 1) and older people (n = 1). The characteristics of included studies are listed in S3 Appendix.

### Barriers and facilitating factors for implementing a preventive intervention

Various categories of barriers and facilitating factors for implementing a preventive intervention were identified, some of which overlapped within the same study (Table 2). The most common types of barriers are family and friends (44/98), limited knowledge (44/98 studies), and economic factors (40/98). The most widely encountered facilitating factors are family and friends (46/98), social support (27/98), and knowledge (15/98). Family and friends, knowledge, and social support are factors that can influence the implementation of a preventive intervention in either direction. Other barriers could not be assigned to any of the eight categories, and appeared in an insufficient number of studies to justify forming additional categories. These include insufficient time, insufficient skills, insufficient motivation/energy, fear (e.g. of needles) in T2DM management or of injuries while being physical active, and travel/transport issues.

**Table 2:** Barriers and facilitating factor categories for implementing a preventive intervention for people with or at risk of T2DM/GDM.

	barriers	facilitating factors	both within a study
Language	22	0	0
economic factors	40	0	0
family and friends	44	46	22
Work	28	0	0
social support	16	27	8
Religion	5	8	1
Culture	30	0	0
knowledge	44	15	4
other barriers (e.g. insufficient time, problems with travelling, insufficient motivation)	9	n.a.	n.a.

GDM: gestational diabetes; n.a.: not applicable; T2DM: type 2 diabetes mellitus

The total number of barriers and facilitating factors within each category is based on the number of identified studies which mentioned this barrier or facilitating factor.

### T2DM/GDM

Most studies were conducted in a T2DM population (88/98), only four studies in the context of GDM. The most common barriers in GDM studies were economic factors (n = 4) and limited knowledge (n = 3). One important facilitating factor for the prevention of GDM is the wellbeing of the unborn child.

### Language

We identified 22/98 studies which describe language barriers. All but one study, performed in an ethnic group [25], were within the vulnerable group of migrants. In most studies, language barriers led to difficulties in understanding either written materials or oral information [26]. In addition, anxiety about dealing with native speakers was reported to be a major barrier to health service access for non-native speakers [27].

### Economic factors

Economic barriers included not only the cost of preventive interventions (e.g. nutritional/fitness courses or travel costs), but more importantly the cost of healthy food. Lack of health insurance also constituted an economic barrier [28]. Economic factors were identified only as barriers; possible facilitating factors such as incentives for participating or insurance bonus programs were not reported in the identified studies.

### Family and friends

Family and friends can act as both barrier and facilitating factor. This depends on whether they act supportively [29] regarding the preventive intervention or obstruct it. For example, family members may not want to change their dietary habits along with the patient, thus making it hard for the patient to stick to their new diet or force them to prepare two different meals [30]. There was also a gender difference in how factors in the family and friends category were perceived. Women often describe family as a barrier because childcare [31] or household chores [32] result in a lack of time to implement preventive interventions.

### Work

Work creates a lack of time in which to join preventive courses or tutorials [33]. Shift work leads to variable work hours, making it difficult to join courses on a regular basis. Additionally, patients working shifts [34] or nights [34, 35] find it harder to eat regular meals.

### Social support

This category describes social support as both a barrier and facilitating factor. Social support in this category encompasses government support, along with covers any other type of external help. For example, single mothers listed insufficient childcare opportunities while participating in a preventive intervention as a barrier [36].

### Religion/culture

Religion and culture were two separate categories of barriers and facilitating factors but overlapped in many ways. For instance, religious festivals often involve a lot of traditional food.

Religious beliefs could be both barrier and facilitating factor. Bhattacharya et al. showed this within the same study [37]. Different options exist on how to interpret God´s will and how to embed T2DM in the religious context. The diagnosis of T2DM could be considered as “God given”, leading believers to “surrender to God´s will” [38, 39]. Another belief was “that not taking care of one´s body goes against (…) self-responsibility” [37]. Some studies demonstrated the importance of rice in many cultures including South Asian, African, and Latino Communities. The preventive diets often referred to western food and therefore gained little acceptance compared to rice-based traditional foods [40].

### Limited knowledge

Limited knowledge describes the inability to implement given information, e.g. in the diet. Most information about food and cooking was designed based on western diets, and therefore leaves patients with unanswered questions regarding meal preparation and food choices [26]. Some participants described the amount of information on diabetes prevention as overwhelming and unrealistic for their daily lives [23, 24].

### Other barriers

Another overarching barrier across groups was insufficient time, due for example to managing family-owned businesses with long workhours, or to family commitments such as childcare [41]. Furthermore, studies described problems with travel distance, e.g. to prevention courses or “food outlets” [42], or travel issues due to elevated age [41]. Insufficient personal motivation [23, 24, 36] was another common barrier.

### Vulnerable groups and gender aspects

Only a single barrier and no facilitating factor was unique for one specific vulnerable group (language barrier for migrants). All other barriers and facilitating factors were identified at least once in each specific vulnerable group. Gender-specific perceptions were identified especially in the category ‘family and friends’. Male T2DM patients see family as a facilitator [43], whereas women more often describe family as a barrier due to childcare issues [36], insufficient diet compliance of the other family members [33], or culturally based reasons like shame of being seen in sportswear or going to the gym [36], or husbands not wanting their wives to go to the gym [44].

## Discussion

In this scoping review, we were able to identify and categorize various barriers and facilitating factors in the prevention of T2DM/GDM in vulnerable groups. Most studies targeted only one vulnerable group, but some targeted mixed groups like “older migrants”. The three most common vulnerable groups within the 98 identified studies were “migrants”, “ethnic groups”, and “people with low socio-economic status”.

All eight categories of barriers and facilitating factors were identified within each vulnerable group apart from one (language barrier for migrants). The most common facilitating factors (family/friends, social support) are similar in each vulnerable group. The most common barriers were limited knowledge, family/friends, and economic factors. The differences between vulnerable groups with respect to te most common barriers were minimal. Only one category of barrier could be attributed to a single vulnerable group only (language barrier to migrants). Factors including insufficient motivation, cost of healthy food, or work are similar to those found in T2DM/GDM prevention designed for general high-risk population [45]. However, cultural or religious factors seem to be exclusive to vulnerable groups, and are important especially for migrants and ethnic groups.

It appears that barriers and facilitating factors between the vulnerable groups are similar, which should be taken into account when developing T2DM/GDM preventive interventions targeted at vulnerable groups. Still, the religious and cultural background of each vulnerable group needs to be considered separately, which may lead to different approaches for T2DM/GDM prevention in each vulnerable group.

Gender differences in the direction of certain factors require that women and men be approached in different ways for T2DM/GDM prevention. For families, lack of social support such as childcare could act as a barrier for parents to join preventive interventions. This could be addressed by providing childcare as part of the intervention, or by allowing children to join the intervention. Men could be further motivated e.g. by integrating their partners into the preventive intervention.

All barriers and facilitating factors identified to be relevant to vulnerable groups should be accounted for, either by adapting existing programs, or by developing new interventions specifically for vulnerable groups. Examples where this has already been implemented are the adaption of the National Diabetes Prevention Program (NDPP) to men with a low socio-economic status [46], and a culturally adapted lifestyle intervention among Iraqi immigrants in Sweden [47]. Straightforward options to modify existing interventions are translations, or adaptation to alternative food cultures such as rice-based diets. To address more complex aspects, such as the influence of family/friends or gender differences, it may be necessary to develop new preventive interventions for the target population.

### Limitations

The publication date of included studies was limited to the past decade, because of the limited time frame available within the “National awareness and prevention strategy on diabetes in Germany”. The effect of this limitation is likely small, because barriers and facilitating factors reported in older studies are likely outdated in view of growing digitization. The eight categories for barriers and facilitating factors were predetermined. Therefore, some factors that did not fit into any category were classified post-hoc as “other barriers”, but it seems possible that alternative or additional categories, may have been relevant. The richness of the collected data warrants additional analyses (e.g. thematic analysis) that could be undertaken in the future, but were beyond the scope of this scoping review.

## Conclusions

We identified various barriers and facilitating factors which should be considered in the development of future preventive interventions and adaption of existing interventions. Some general barriers and facilitating factors should be considered regardless of the presence of vulnerable groups, including economic factors and gender aspects. Religious and cultural factors, in particular, require different approaches for each vulnerable group.

## Supporting information

S1 Appendix Search strategy. (DOCX)

S2 Appendix Excluded studies. (DOCX)

S3 Appendix Characteristics of the included studies. (DOCX)

## Abbreviations

GDM: Gestational diabetes mellitus
PPC: Population, Concept, Context
T2DM: Type 2 diabetes mellitus
WHO: World Health Organization
