# Supplementary material for: Barriers and facilitating factors in the prevention of diabetes type 2 and gestational diabetes in vulnerable groups: A scoping review
Source: PLoS One. 2020 May 13;15(5):e0232250. doi: 10.1371/journal.pone.0232250 (PMC7219729; doi:10.1371/journal.pone.0232250)
Supplement: S1 Appendix — (DOCX) [file pone.0232250.s001.docx]

**Supplement 1: Search strategies**

**Table 1: Search strategy for PubMed, September 2019**

|  | "Diabetes, Gestational"[Mesh] OR "Diabetes Mellitus, Type 2"[Mesh] OR gestational diabetes[tiab] OR diabetes mellitus, gestational[tiab] OR pregnancy-induced diabetes[tiab] OR type 2 diabetes[tiab] OR “diabetes mellitus type II”[tiab] OR type 2 diabetes mellitus[tiab] OR “diabetes type 2”[tiab] |
| --- | --- |
| AND | ("Ethnic Groups"[Mesh] OR "Minority Groups"[Mesh] OR "Poverty Areas"[Mesh] OR "Vulnerable Populations"[Mesh] OR "Health Status Disparities"[Mesh] OR "Cultural Diversity"[Mesh] OR "Socioeconomic Factors"[Mesh] OR "Aged"[Mesh] OR "Substance-Related Disorders"[Mesh] OR "Malnutrition"[Mesh] OR "Disabled Persons"[Mesh] OR "Educational Status"[Mesh] OR "Emigrants and Immigrants"[Mesh] OR "Homeless Persons"[Mesh] OR "Minors"[Mesh] OR "Transients and Migrants"[Mesh] OR "Refugees"[Mesh] OR "Unemployment"[Mesh] OR "Mental Disorders"[Mesh] OR ethnic group*[tiab] OR ethnic population*[tiab] OR minority[tiab] OR minorities[tiab] OR ethnic minorit*[tiab] OR poverty[tiab] OR destitution[tiab] OR poor housing[tiab] OR addiction[tiab] OR drug abuse[tiab] OR malnutrition[tiab] OR malnourished[tiab] OR vulnerable population*[tiab] OR vulnerable group*[tiab] OR socioeconomic factor*[tiab] OR socioeconomic aspect*[tiab] OR deprived[tiab] OR health status[tiab] OR aged[tiab] OR elderly[tiab] OR elders[tiab] OR minors[tiab] OR disabled[tiab] OR disability[tiab] OR level of education[tiab] OR education level[tiab] OR mental disorder[tiab] OR need for care[tiab] OR need of care[tiab] OR care dependency[tiab] OR unemployment[tiab] OR ethnic disparity[tiab] OR ethnic disparities[tiab] OR migrant[tiab] OR migrants[tiab] OR immigrant[tiab] OR immigrants[tiab] OR asylum[tiab] OR refugees[tiab] OR cultural diversity[tiab] OR multicultural aspect*[tiab] OR multicultural factor*[tiab] OR religion[tiab] OR homeless*[tiab]) |
| AND | (“Primary Prevention"[Mesh] OR "Secondary Prevention"[Mesh] OR "Tertiary Prevention"[Mesh] OR "Preventive Health Services"[Mesh] OR "Mass Screening"[Mesh] OR "Health Promotion"[Mesh] OR "Health Education"[Mesh] OR "Patient Education as Topic"[Mesh] OR "Health Literacy"[Mesh] OR "Health Services for Persons with Disabilities"[Mesh] OR "Health Services for the Aged"[Mesh] OR "Health Services, Indigenous"[Mesh] OR "Culturally Competent Care"[Mesh] OR prevention[tiab] OR prevent[tiab] OR preventing[tiab] OR health service*[tiab] OR screening[tiab] OR health promotion[tiab] OR health education[tiab] OR patient education[tiab] OR health literacy[tiab] OR health care[tiab]) |
| AND | ("Health Communication"[Mesh] OR "Reminder Systems"[Mesh] OR "Counseling"[Mesh] OR "Communications Media"[Mesh] OR "Motivation"[Mesh] OR "Information Dissemination"[Mesh] OR "Consumer Health Information"[Mesh] OR "Pamphlets"[Mesh] OR "Information Literacy"[Mesh] OR "Teaching Materials"[Mesh] OR intervention[tiab] OR interventions[tiab] OR health communication[tiab] OR communication media[tiab] OR reminder system*[tiab] OR counseling[tiab] OR counselling[tiab] OR health information[tiab] OR information dissemination[tiab] OR information literacy[tiab] OR teaching material*[tiab] OR pamphlet[tiab] OR pamphlets[tiab] OR booklet[tiab] OR booklets[tiab] OR leaflet[tiab] OR leaflets[tiab] OR flyer[tiab] OR flyers[tiab] OR poster[tiab] OR posters[tiab] OR brochure[tiab] OR brochures[tiab] OR access[tiab] OR communication strategy[tiab] OR communication strategies[tiab] OR strategy[tiab] OR strategies[tiab] OR audio*[tiab] OR video[tiab] OR videos[tiab] OR dvd*[tiab] OR compact disc*[tiab] OR cd[tiab] OR cds[tiab] OR "Multimedia"[Mesh] OR multimedia[tiab] OR multi-media[tiab] OR "Telecommunications"[Mesh] OR "Internet"[Mesh] OR internet[tiab] OR web[tiab] OR website*[tiab] OR online[tiab] OR electronic mail*[tiab] OR email*[tiab] OR mail*[tiab] OR "Blogging"[Mesh] OR blog*[tiab] OR weblog*[tiab] OR podcast*[tiab] OR portal*[tiab] OR computer program*[tiab] OR computer mediated[tiab] OR computer based[tiab] OR computer assisted[tiab] OR "Correspondence as Topic"[Mesh] OR telephon*[tiab] OR phone[tiab] OR phones[tiab] OR text messag*[tiab] OR sms[tiab] OR facilitator[tiab] OR facilitators[tiab] OR facilitate[tiab] OR motivation[tiab] OR motivators[tiab] OR motivational strategy[tiab] OR motivational strategies[tiab] OR enablers[tiab] OR promotional[tiab] OR beneficial[tiab] OR helpful[tiab] OR fostering[tiab] OR advantageous[tiab] OR barrier[tiab] OR barriers[tiab] OR barricade[tiab] OR impeding[tiab] OR hindering[tiab]) |
| AND | („2008/01/01“[EDAT] : „3000“[EDAT]) |

**Table 2: Search strategy for Embase, September 2019**

|  | 'non insulin dependent diabetes mellitus'/exp OR 'pregnancy diabetes mellitus'/exp OR (“gestational diabetes” OR “diabetes mellitus, gestational” OR “pregnancy-induced diabetes” OR “type 2 diabetes” OR “diabetes mellitus type II” OR “type 2 diabetes mellitus” OR “diabetes type 2”):ti,ab,kw |
| --- | --- |
| AND | ('ethnic group'/exp OR 'minority group'/exp OR 'poverty'/exp OR 'vulnerable population'/exp OR 'health disparity'/exp OR 'cultural diversity'/exp OR 'socioeconomics'/exp OR 'aged'/exp OR 'drug dependence'/exp OR 'malnutrition'/exp OR 'disabled person'/exp OR 'educational status'/exp OR 'migrant'/exp OR 'homelessness'/exp OR 'homeless person'/exp OR 'minor (person)'/exp OR 'refugee'/exp OR 'unemployment'/exp OR 'mental disease'/exp OR (“ethnic group*” OR “ethnic population*” OR minority OR minorities OR “ethnic minorit*” OR poverty OR destitution OR “poor housing” OR addiction OR “drug abuse” OR malnutrition OR malnourished OR “vulnerable population*” OR “vulnerable group*” OR “socioeconomic factor*” OR “socioeconomic aspect*” OR “low socioeconomic status” OR deprived OR “health status” OR aged OR elderly OR elders OR minors OR disabled OR disability OR “level of education” OR “education level” OR “low level of education” OR “mental disorder” OR “need for care” OR “need of care” OR “care dependency” OR unemployment OR “ethnic disparity” OR “ethnic disparities” OR migrant OR migrants OR immigrant OR immigrants OR asylum OR refugees OR “cultural diversity” OR “multicultural aspect*” OR “multicultural factor*” OR religion OR homeless*):ab,ti,kw) |
| AND | ('primary prevention'/exp OR 'secondary prevention'/exp OR 'tertiary prevention'/exp OR 'preventive health service'/exp OR 'mass screening'/exp OR 'health promotion'/exp OR 'health education'/exp OR 'patient education'/exp OR 'health literacy'/exp OR 'health service'/exp OR 'elderly care'/exp OR 'indigenous health care'/exp OR 'transcultural care'/exp OR (prevention OR prevent OR preventing OR “health service*” OR screening OR “health promotion” OR “health education” OR “patient education” OR “health literacy” OR “health care”):ti,ab,kw) |
| AND | ('medical information'/exp OR 'reminder system'/exp OR 'counseling'/exp OR 'mass medium'/exp OR 'motivation'/exp OR 'information dissemination'/exp OR 'consumer health information'/exp OR 'publication'/exp OR 'information literacy'/exp OR 'teaching'/exp OR 'multimedia'/exp OR 'telecommunication'/exp OR 'Internet'/exp OR 'blogging'/exp OR (intervention OR interventions OR “health communication” OR “communication media” OR “reminder system*” OR counseling OR counselling OR “health information” OR “information dissemination” OR “information literacy” OR “teaching material*” OR pamphlet OR pamphlets OR booklet OR booklets OR leaflet OR leaflets OR flyer OR flyers OR poster OR posters OR brochure OR brochures OR access OR “communication strategy” OR “communication strategies” OR strategy OR strategies OR audio* OR video OR videos OR dvd* OR “compact disc*” OR cd OR cds OR multimedia OR “multi-media” OR “media campaign” OR internet OR web OR website* OR online OR “electronic mail*” OR email* OR mail* OR blog* OR weblog* OR podcast* OR portal* OR “computer program*” OR “computer mediated” OR “computer based” OR “computer assisted” OR telephon* OR phone OR phones OR “text messag*” OR sms OR facilitator OR facilitators OR facilitate OR motivation OR motivators OR “motivational strategy” OR “motivational strategies” OR enablers OR promotional OR beneficial OR helpful OR fostering OR advantageous OR barrier OR barriers OR barricade OR impeding OR hindering):ti,ab,kw) |
| AND | ([embase]/lim) |
| NOT | (('comment' OR 'letter' OR 'editorial'):it) |
| AND | ([1-1-2008]/sd NOT [1-1-3000]/sd) |
| AND | (embase NOT (embase AND medline)) |

**Table 3: Search strategy for PsycINFO, September 2019**

|  | DE "Gestational Diabetes" OR DE "Type 2 Diabetes" OR TI (gestational diabetes OR diabetes mellitus, gestational OR pregnancy-induced diabetes OR type 2 diabetes OR “diabetes mellitus type II” OR type 2 diabetes mellitus OR “diabetes type 2”) OR AB (gestational diabetes OR diabetes mellitus, gestational OR pregnancy-induced diabetes OR type 2 diabetes OR “diabetes mellitus type II” OR type 2 diabetes mellitus OR “diabetes type 2”) |
| --- | --- |
| AND | DE "Racial and Ethnic Groups" OR DE "Racial and Ethnic Differences" OR DE "Minority Groups" OR DE "Health Disparities" OR DE "Poverty" OR DE "Poverty Areas" OR DE "Social Deprivation" OR DE "Human Migration" OR DE "Socioeconomic Status" OR DE "Aged (Attitudes Toward)" OR DE "Addiction" OR DE "Substance Abuse and Addiction Measures" OR DE "Nutritional Deficiencies" OR DE "Disabled (Attitudes Toward)" OR DE "Immigration" OR DE "Homeless" OR DE "Refugees" OR DE "Unemployment" OR DE "Mental Disorders" OR TI (ethnic group* OR ethnic population* OR minorit* OR ethnic minorit* OR poverty OR destitution OR poor housing OR addiction OR drug abuse OR malnutrition OR malnourished OR vulnerable population* OR vulnerable group* OR socioeconomic factor* OR socioeconomic aspect* OR low socioeconomic status OR deprived OR health status OR aged OR elderly OR elders OR minors OR disabled OR disability OR level of education OR education level OR low level of education OR mental disorder OR need for care OR need of care OR care dependency OR unemployment OR ethnic disparit* OR migrant* OR immigrant* OR asylum OR refugees OR cultural diversity OR multicultural aspect* OR multicultural factor* OR religion OR homeless*) OR AB (ethnic group* OR ethnic population* OR minorit* OR ethnic minorit* OR poverty OR destitution OR poor housing OR addiction OR drug abuse OR malnutrition OR malnourished OR vulnerable population* OR vulnerable group* OR socioeconomic factor* OR socioeconomic aspect* OR low socioeconomic status OR deprived OR health status OR aged OR elderly OR elders OR minors OR disabled OR disability OR level of education OR education level OR low level of education OR mental disorder OR need for care OR need of care OR care dependency OR unemployment OR ethnic disparit* OR migrant* OR immigrant* OR asylum OR refugees OR cultural diversity OR multicultural aspect* OR multicultural factor* OR religion OR homeless*) |
| AND | DE "Prevention" OR DE "Health Care Services" OR DE "Public Health Services" OR DE "Health Literacy" OR DE "Primary Health Care" OR DE "Health Promotion" OR DE "Health Education" OR DE "Health Screening" OR TI (prevention OR prevent OR preventing OR health service* OR screening OR health promotion OR health education OR patient education OR health literacy OR health care) OR AB (prevention OR prevent OR preventing OR health service* OR screening OR health promotion OR health education OR patient education OR health literacy OR health care) |
| AND | DE "Manual Communication" OR DE "Communications Media" OR DE "Printed Communications Media" OR DE "Electronic Communication" OR DE "Audiovisual Communications Media" OR DE "Counseling" OR DE "Motivation" OR DE "Information Dissemination" OR DE "Consumer Education" OR DE "Information Literacy" OR DE "Multimedia" OR DE "Telecommunications Media" OR DE "Internet" OR DE "Blog" TI (intervention* OR health communication OR communication media OR reminder system* OR counse#ing OR health information OR information dissemination OR information literacy OR teaching material* OR pamphlet* OR booklet* OR leaflet* OR flyer* OR poster* OR brochure* OR access OR communication strateg* OR strateg* OR audio* OR video OR videos OR dvd* OR compact disc* OR cd OR cds OR multimedia OR multi-media OR media campaign OR internet OR web OR website* OR online OR electronic mail* OR email* OR mail* OR blog* OR weblog* OR podcast* OR portal* OR computer program* OR computer mediated OR computer based OR computer assisted OR telephon* OR phone OR phones OR text messag* OR sms OR facilitator* OR facilitate OR motivation OR motivators OR motivational strategy* OR enablers OR promotional OR beneficial OR helpful OR fostering OR advantageous OR barrier* OR barricade OR impeding OR hindering) OR AB (intervention* OR health communication OR communication media OR reminder system* OR counse#ing OR health information OR information dissemination OR information literacy OR teaching material* OR pamphlet* OR booklet* OR leaflet* OR flyer* OR poster* OR brochure* OR access OR communication strateg* OR strateg* OR audio* OR video OR videos OR dvd* OR compact disc* OR cd OR cds OR multimedia OR multi-media OR media campaign OR internet OR web OR website* OR online OR electronic mail* OR email* OR mail* OR blog* OR weblog* OR podcast* OR portal* OR computer program* OR computer mediated OR computer based OR computer assisted OR telephon* OR phone OR phones OR text messag* OR sms OR facilitator* OR facilitate OR motivation OR motivators OR motivational strateg* OR enablers OR promotional OR beneficial OR helpful OR fostering OR advantageous OR barrier* OR barricade OR impeding OR hindering) |
|  | 2008-2018 |

**Table 4: Search strategy for PSYNDEX, September 2019**

|  | DE "Gestational Diabetes" OR DE "Type 2 Diabetes" OR DE "Blood Sugar" OR TI (gestational diabetes OR diabetes mellitus, gestational OR pregnancy-induced diabetes OR type 2 diabetes OR “diabetes mellitus type II” OR type 2 diabetes mellitus OR “diabetes type 2”) OR AB (gestational diabetes OR diabetes mellitus, gestational OR pregnancy-induced diabetes OR type 2 diabetes OR “diabetes mellitus type II” OR type 2 diabetes mellitus OR “diabetes type 2”) |
| --- | --- |
| AND | DE "Racial and Ethnic Groups" OR DE "Racial and Ethnic Differences" OR DE "Minority Groups" OR DE "Health Disparities" OR DE "Poverty" OR DE "Poverty Areas" OR DE "Social Deprivation" OR DE "Human Migration" OR DE "Socioeconomic Status" OR DE "Aged (Attitudes Toward)" OR DE "Addiction" OR DE "Substance Abuse and Addiction Measures" OR DE "Nutritional Deficiencies" OR DE "Disabled (Attitudes Toward)" OR DE "Immigration" OR DE "Homeless" OR DE "Refugees" OR DE "Unemployment" OR DE "Mental Disorders" OR TI (ethnic group* OR ethnic population* OR minorit* OR ethnic minorit* OR poverty OR destitution OR poor housing OR addiction OR drug abuse OR malnutrition OR malnourished OR vulnerable population* OR vulnerable group* OR socioeconomic factor* OR socioeconomic aspect* OR low socioeconomic status OR deprived OR health status OR aged OR elderly OR elders OR minors OR disabled OR disability OR level of education OR education level OR low level of education OR mental disorder OR need for care OR need of care OR care dependency OR unemployment OR ethnic disparit* OR migrant* OR immigrant* OR asylum OR refugees OR cultural diversity OR multicultural aspect* OR multicultural factor* OR religion OR homeless*) OR AB (ethnic group* OR ethnic population* OR minorit* OR ethnic minorit* OR poverty OR destitution OR poor housing OR addiction OR drug abuse OR malnutrition OR malnourished OR vulnerable population* OR vulnerable group* OR socioeconomic factor* OR socioeconomic aspect* OR low socioeconomic status OR deprived OR health status OR aged OR elderly OR elders OR minors OR disabled OR disability OR level of education OR education level OR low level of education OR mental disorder OR need for care OR need of care OR care dependency OR unemployment OR ethnic disparit* OR migrant* OR immigrant* OR asylum OR refugees OR cultural diversity OR multicultural aspect* OR multicultural factor* OR religion OR homeless*) |
| AND | DE "Prevention" OR DE "Health Care Services" OR DE "Public Health Services" OR DE "Health Literacy" OR DE "Primary Health Care" OR DE "Health Promotion" OR DE "Health Education" OR DE "Health Screening" OR TI (prevention OR prevent OR preventing OR health service* OR screening OR health promotion OR health education OR patient education OR health literacy OR health care) OR AB (prevention OR prevent OR preventing OR health service* OR screening OR health promotion OR health education OR patient education OR health literacy OR health care) |
| AND | DE "Manual Communication" OR DE "Communications Media" OR DE "Printed Communications Media" OR DE "Electronic Communication" OR DE "Audiovisual Communications Media" OR DE "Counseling" OR DE "Motivation" OR DE "Information Dissemination" OR DE "Consumer Education" OR DE "Information Literacy" OR DE "Multimedia" OR DE "Telecommunications Media" OR DE "Internet" OR DE "Blog" TI (intervention* OR health communication OR communication media OR reminder system* OR counse#ing OR health information OR information dissemination OR information literacy OR teaching material* OR pamphlet* OR booklet* OR leaflet* OR flyer* OR poster* OR brochure* OR access OR communication strateg* OR strateg* OR audio* OR video OR videos OR dvd* OR compact disc* OR cd OR cds OR multimedia OR multi-media OR media campaign OR internet OR web OR website* OR online OR electronic mail* OR email* OR mail* OR blog* OR weblog* OR podcast* OR portal* OR computer program* OR computer mediated OR computer based OR computer assisted OR telephon* OR phone OR phones OR text messag* OR sms OR facilitator* OR facilitate OR motivation OR motivators OR motivational strategy* OR enablers OR promotional OR beneficial OR helpful OR fostering OR advantageous OR barrier* OR barricade OR impeding OR hindering) OR AB (intervention* OR health communication OR communication media OR reminder system* OR counse#ing OR health information OR information dissemination OR information literacy OR teaching material* OR pamphlet* OR booklet* OR leaflet* OR flyer* OR poster* OR brochure* OR access OR communication strateg* OR strateg* OR audio* OR video OR videos OR dvd* OR compact disc* OR cd OR cds OR multimedia OR multi-media OR media campaign OR internet OR web OR website* OR online OR electronic mail* OR email* OR mail* OR blog* OR weblog* OR podcast* OR portal* OR computer program* OR computer mediated OR computer based OR computer assisted OR telephon* OR phone OR phones OR text messag* OR sms OR facilitator* OR facilitate OR motivation OR motivators OR motivational strateg* OR enablers OR promotional OR beneficial OR helpful OR fostering OR advantageous OR barrier* OR barricade OR impeding OR hindering) |
|  | 2008-2018 |

**Table 5: Search strategy for SSCI, September 2019**

|  | TS=(gestational diabetes OR diabetes mellitus, gestational OR pregnancy-induced diabetes OR type 2 diabetes OR “diabetes mellitus type II” OR type 2 diabetes mellitus OR “diabetes type 2”) |
| --- | --- |
| AND | (TS=(ethnic group* OR ethnic population* OR minorit* OR ethnic minorit* OR poverty OR destitution OR poor housing OR addiction OR drug abuse OR malnutrition OR malnourished OR vulnerable population* OR vulnerable group* OR socioeconomic factor* OR socioeconomic aspect* OR “low socioeconomic status” OR deprived OR health status OR aged OR elderly OR elders OR minors OR disabled OR disability OR “level of education” OR education level OR “low level of education” OR mental disorder OR “need for care” OR “need of care” OR care dependency OR unemployment OR ethnic disparit* OR migrant* OR immigrant* OR asylum OR refugees OR cultural diversity OR multicultural aspect* OR multicultural factor* OR religion OR homeless*)) |
| AND | (TS=(prevention OR prevent OR preventing OR health service* OR screening OR health promotion OR health education OR patient education OR health literacy OR health care)) |
| AND | (TS=(intervention* OR health communication OR communication media OR reminder system* OR counse$ing OR health information OR information dissemination OR information literacy OR teaching material* OR pamphlet* OR booklet* OR leaflet* OR flyer* OR poster* OR brochure* OR access OR communication strateg* OR strateg* OR audio* OR video OR videos OR dvd* OR compact disc* OR cd OR cds OR multimedia OR multi-media OR media campaign OR internet OR web OR website* OR online OR electronic mail* OR email* OR mail* OR blog* OR weblog* OR podcast* OR portal* OR computer program* OR computer mediated OR computer based OR computer assisted OR telephon* OR phone OR phones OR text messag* OR sms OR facilitator* OR facilitate OR motivation OR motivators OR motivational strategy* OR enablers OR promotional OR beneficial OR helpful OR fostering OR advantageous OR barrier* OR barricade OR impeding OR hindering)) |
|  | 2008-2018 |

**Table 6: Search strategy for CINAHL, September 2019**

|  | MH "Diabetes Mellitus, Gestational" OR MH "Diabetes Mellitus, Type 2" OR TI (gestational diabetes OR diabetes mellitus, gestational OR pregnancy-induced diabetes OR type 2 diabetes OR “diabetes mellitus type II” OR type 2 diabetes mellitus OR “diabetes type 2”) OR AB (gestational diabetes OR diabetes mellitus, gestational OR pregnancy-induced diabetes OR type 2 diabetes OR “diabetes mellitus type II” OR type 2 diabetes mellitus OR “diabetes type 2”) |
| --- | --- |
| AND | MH "Ethnic Groups" OR MH "Minority Groups" OR MH "Poverty Areas" OR MH "Vulnerability" OR MH "Health Status Disparities" OR MH "Cultural Diversity" OR MH "Socioeconomic Factors" OR MH "Aged" OR MH "Substance Use Disorders" OR MH "Malnutrition" OR MH "Disabled" OR MH "Mentally Disabled Persons" OR MH "Educational Status" OR MH "Immigrants" OR MH "Homelessness" OR MH "Homeless Persons" OR MH "Minors (Legal)" OR MH "Transients and Migrants" OR MH "Refugees" OR MH "Unemployment" OR MH "Mental Disorders" OR TI (“ethnic group*” OR “ethnic population*” OR minorit* OR “ethnic minorit*” OR poverty OR destitution OR “poor housing” OR addiction OR “drug abuse” OR malnutrition OR malnourished OR “vulnerable population*” OR “vulnerable group*” OR “socioeconomic factor*” OR “socioeconomic aspect*” OR “low socioeconomic status” OR deprived OR “health status” OR aged OR elderly OR elders OR minors OR disabled OR disability OR “level of education” OR “education level” OR “low level of education” OR “mental disorder” OR “need for care” OR “need of care” OR “care dependency” OR unemployment OR “ethnic disparit*” OR migrant* OR immigrant* OR asylum OR refugees OR “cultural diversity” OR “multicultural aspect*” OR “multicultural factor*” OR religion OR homeless*) OR AB (“ethnic group*” OR “ethnic population*” OR minorit* OR “ethnic minorit*” OR poverty OR destitution OR “poor housing” OR addiction OR “drug abuse” OR malnutrition OR malnourished OR “vulnerable population*” OR “vulnerable group*” OR “socioeconomic factor*” OR “socioeconomic aspect*” OR “low socioeconomic status” OR deprived OR “health status” OR aged OR elderly OR elders OR minors OR disabled OR disability OR “level of education” OR “education level” OR “low level of education” OR “mental disorder” OR “need for care” OR “need of care” OR “care dependency” OR unemployment OR “ethnic disparit*” OR migrant* OR immigrant* OR asylum OR refugees OR “cultural diversity” OR “multicultural aspect*” OR “multicultural factor*” OR religion OR homeless*) |
| AND | MH "Preventive Health Care" OR MH "Health Services Needs and Demand" OR MH "Health Services for the Aged" OR MH "Health Services for Persons with Disabilities" OR MH "Health Services, Indigenous" OR MH "Health Screening" OR MH "Health Promotion" OR MH "Health Education" OR MH "Health Literacy" OR TI (prevention OR prevent OR preventing OR “health service*” OR screening OR “health promotion” OR “health education” OR “patient education” OR “health literacy” OR “health care”) OR AB (prevention OR prevent OR preventing OR “health service*” OR screening OR “health promotion” OR “health education” OR “patient education” OR “health literacy” OR “health care”) |
| AND | MH "Health Care Delivery OR MH "Reminder Systems" OR MH "Counseling" OR MH "Communications Media" OR MH "Social Media" OR MH "Motivation" OR MH "Information Literacy" OR MH "Home Health Care Information Systems" OR MH "Consumer Health Information" OR MH "Pamphlets" OR MH "Teaching Materials" OR MH "Multimedia" OR MH "Telecommunications" OR MH "Internet" OR MH "Blogs" OR TI (intervention OR interventions OR “health communication” OR “communication media” OR “reminder system*” OR counse#ing OR “health information” OR “information dissemination” OR “information literacy” OR “teaching material*” OR pamphlet* OR booklet* OR leaflet* OR flyer* OR poster* OR brochure* OR access OR “communication strateg* OR strateg* OR audio* OR video OR videos OR dvd* OR compact disc* OR cd OR cds OR multimedia OR “multi-media” OR “media campaign” OR internet OR web OR website* OR online OR “electronic mail*” OR email* OR mail* OR blog* OR weblog* OR podcast* OR portal* OR “computer program*” OR “computer mediated” OR “computer based” OR “computer assisted” OR telephon* OR phone OR phones OR “text messag*” OR sms OR facilitator* OR facilitate OR motivation OR motivators OR “motivational strategy*” OR enablers OR promotional OR beneficial OR helpful OR fostering OR advantageous OR barrier OR barriers OR barricade OR impeding OR hindering) OR AB (intervention OR interventions OR “health communication” OR “communication media” OR “reminder system*” OR counse#ing OR “health information” OR “information dissemination” OR “information literacy” OR “teaching material*” OR pamphlet* OR booklet* OR leaflet* OR flyer* OR poster* OR brochure* OR access OR “communication strateg* OR strateg* OR audio* OR video OR videos OR dvd* OR compact disc* OR cd OR cds OR multimedia OR “multi-media” OR “media campaign” OR internet OR web OR website* OR online OR “electronic mail*” OR email* OR mail* OR blog* OR weblog* OR podcast* OR portal* OR “computer program*” OR “computer mediated” OR “computer based” OR “computer assisted” OR telephon* OR phone OR phones OR “text messag*” OR sms OR facilitator* OR facilitate OR motivation OR motivators OR “motivational strategy*” OR enablers OR promotional OR beneficial OR helpful OR fostering OR advantageous OR barrier OR barriers OR barricade OR impeding OR hindering) |
|  | 2008-2018 |
