# Supplementary material for: Barriers and facilitating factors in the prevention of diabetes type 2 and gestational diabetes in vulnerable groups: A scoping review
Source: PLoS One. 2020 May 13;15(5):e0232250. doi: 10.1371/journal.pone.0232250 (PMC7219729; doi:10.1371/journal.pone.0232250)
Supplement: S3 Appendix — (DOCX) [file pone.0232250.s003.docx]

Supplement 3: Characteristics of the included studies

| Study | Methods | Participants n | Gender [m/f]n(%) | Age[years] mean//range//  n(%) | Vulnerable Group categories | T2DM/  GDM |
| --- | --- | --- | --- | --- | --- | --- |
| Aguayo-Mazzucato C, Diaque P, Hernandez S, Rosas S, Kostic A, Caballero AE. Understanding the growing epidemic of type 2 diabetes in the Hispanic population living in the United States. Diabetes Metab Res Rev. 2019;35(2):e3097. | Review | n.a. | n.a. | n.a. | n.a. | n.a. |
| Alzubaidi H, Mc Namara K, Browning C. Time to question diabetes self-management support for Arabic-speaking migrants: exploring a new model of care. Diabetic medicine: a journal of the British Diabetic Association. 2017;34(3):348-55.  Alzubaidi H, Mc Namara K, Browning C, Marriott J. Barriers and enablers to healthcare access and use among Arabic-speaking and Caucasian English-speaking patients with type 2 diabetes mellitus: a qualitative comparative study. BMJ open. 2015;5(11):e008687. | Interviews and Group-Interviews | 60 | 22(37)/38(63) | 57 | Migrants | T2DM |
| Amirehsani KA. Mexican Americans with type 2 diabetes in an emerging Latino community: Evaluation of health disparity factors and interventions. Home Health Care Management & Practice. 2010;22(7):470-8. | Systematic Review | n.a. | n.a. | n.a. | n.a. | n.a. |
| Aweko J, De Man J, Absetz P, Ostenson CG, Peterson SS, Alvesson HM, et al. Patient and Provider Dilemmas of Type 2 Diabetes Self-Management: A Qualitative Study in Socioeconomically Disadvantaged Communities in Stockholm. International Journal of Environmental Research and Public Health. 2018;15(9). | Interviews and Group interviews | 12 | 6(50)/6(50) | 35–59 6(50)  >60 6(50) | Migrants | T2DM |
| Baig AA, Locklin CA, Foley E, Ewigman B, Meltzer DO, Huang ES. THE ASSOCIATION OF ENGLISH ABILITY AND GLYCEMIC CONTROL AMONG LATINOS WITH DIABETES. Ethnicity & disease. 2014;24(1):28-34. | Systematic Review | n.a. | n.a. | n.a. | n.a. | n.a. |
| Bandyopadhyay M, Small R, Davey M, Oats J, Forster D, Aylward A. Lived experience of gestational diabetes mellitus among immigrant South Asian women in Australia. Aust N Z J Obstet Gynaecol 2011; 51: 360–364. | Interview | 17 | 0(0)/17(100) | 28 | Migrants | GDM |
| Bernstein AM, Rudd N, Gendy G, Moffett K, Adams J, Steele S, et al. Beliefs About Preventive Care, Individual Health, and Lifestyle Change Among Low-Income African American Women at Risk for Diabetes. Holistic Nursing Practice. 2014;28(1):24-30. | Focus Group | 18 | 0(0)/18(100) | 18-24 1(5) 25-29 2(11) 30-34 0(0) 35-39 3(17) 40-44 4(22) 45-49 2(11) 50-54 2(11) 55-59 2(11) 60-64 2(11) | Ethnic Group | T2DM |
| Bertero C, Hjelm K. Social support as described by foreign-born persons diagnosed with type 2 diabetes mellitus and living in Sweden. Nursing & health sciences. 2010;12(4):507-14. | Interview | 34 | 24(29)/10(71) | 56.7 | Ethnic Group | T2DM |
| Bhattacharya G. Spirituality and Type 2 Diabetes Self-Management Among African Americans in the Arkansas Delta. Journal of Social Service Research. 2013;39(4):469-82. | Interview | 31 | 16(52)/15(48) | 45-64 | Ethnic Group | T2DM |
| Blanks SH, Treadwell H, Bazzell A, Graves W, Osaji O, Dean J, et al. Community Engaged Lifestyle Modification Research: Engaging Diabetic and Prediabetic African American Women in Community-Based Interventions. Journal of obesity. 2016;2016:3609289. | Intervention | 79 | 0(0)/79(100) | 29 | Ethnic Group | T2DM |
| Brathwaite AC, Lemonde M. Health Beliefs and Practices of African Immigrants in Canada. Clinical nursing research. 2016;25(6):626-45. | Focus Group | 14 | 3(21.4)/11(78.6) | 44.6 | Migrants | T2DM |
| Brathwaite AC, Lemonde M. Exploring Health Beliefs and Practices of Caribbean Immigrants in Ontario to Prevent Type 2 Diabetes. Journal of transcultural nursing : official journal of the Transcultural Nursing Society. 2017;28(1):15-23. | Focus Group and Questionnaire | 15 | 0(0)/15(100) | 52 | Migrants | T2DM |
| Brown SA, García AA, Steinhardt MA, Guevara H, Moore C, Brown A, et al. Culturally Tailored Diabetes Prevention in the Workplace: Focus Group Interviews With Hispanic Employees. Diabetes Educator. 2015;41(2):175-83. | Focus Group | 36 | 7(19.5)/29(80.5) | 50.4 | Ethnic Group | T2DM |
| Brown SA, Hanis CL. Lessons Learned from 20 Years of Diabetes Self-Management Research With Mexican Americans in Starr County, Texas. The Diabetes educator. 2014;40(4):476-87. | Focus Group | N.R. | N.R. | N.R. | Ethnic Group | T2DM |
| Brunk DR, Taylor AG, Williams IC, Cox DJ, Clark ML. A Culturally Appropriate Self-Management Program for Hispanic Adults With Type 2 Diabetes and Low Health Literacy Skills. Journal of Transcultural Nursing. 2017;28(2):187-94. | Focus Group | 9 | N.R. | 30-66 | Ethnic Group | T2DM |
| Carolan-Olah M, Cassar A. The Experiences of Older Italian Migrants With Type 2 Diabetes: A Qualitative Study. Journal of Transcultural Nursing. 2018;29(2):172-9. | Focus Group | 13 | 8(61.5)/5(38.5) | 68-85 | Older migrants | T2DM |
| Carolan-Olah MC, Cassar A, Quiazon R, Lynch S. Diabetes care and service access among elderly Vietnamese with type 2 diabetes. BMC health services research. 2013;13:447. | Focus Group | 15 | 4(27)/11(73) | 60-69 4(27) ≥70 11 (73) | Older migrants | T2DM |
| Castro-Rivas E, Boutin-Foster C, Milan M, Kanna B. "Es como uno bomba de tiempo [It's like a time bomb]": A qualitative analysis of perceptions of diabetes among first-degree relatives of latino patients with diabetes. Diabetes Spectrum. 2014;27(1):50-7. | Focus Group | 23 | 15(65)/8(35) | 46.39 | Ethnic Group | T2DM |
| Cha E, Yang K, Lee J, Min J, Kim KH, Dunbar SB, et al. Understanding cultural issues in the diabetes self-management behaviors of Korean immigrants. The Diabetes educator. 2012;38(6):835-44. | Interview | 20 | 9(45)/11(55) | 64.5 | Migrants | T2DM |
| Chang C, Khurana S, Strodel R, Camp A, Magenheimer E, Hawley N. Perceived Barriers to Physical Activity Among Low-Income Latina Women at Risk for Type 2 Diabetes. Diabetes Educator. 2018;44(5):444-53. | Questionnaire | 160 | 0(0)/160(100) | 33.7 | Migrants | T2DM |
| Chaufan C, Constantino S, Davis M. ‘It's a full time job being poor’: understanding barriers to diabetes prevention in immigrant communities in the USA. Critical Public Health. 2012;22(2):147-58. | Focus Group | 15 | N.R. | N.R. | Migrants | T2DM |
| Chlebowy DO, Hood S, LaJoie AS. Facilitators and barriers to self-management of type 2 diabetes among urban African American adults: focus group findings. The Diabetes educator. 2010;36(6):897-905. | Focus Group | 38 | 11(29)/27(71) | 66(44-87) | Ethnic Group | T2DM |
| Chesla C, Chun KM, Kwan CML. Cultural and family challenges to managing type 2 diabetes in immigrant Chinese Americans Diabetes Care. 2009;32(10):1812-1816 | Interview | 40 | N.R. | 62 | Migrants | T2DM |
| Choi SE, Lee JJ, Park JJ, Sarkisian CA. Spousal Support in Diabetes Self-Management Among Korean Immigrant Older Adults. Research in gerontological nursing. 2015;8(2):94-104. | Focus Group | 33 (16 T2DM/17 Spouses) | T2DM 8(50)/8(50) Spouses 9(53)/8(47) | T2DM 68.1 Spouses 74.4 | Migrants | T2DM |
| Choi TST, Walker KZ, Palermo C. Diabetes management in a foreign land: A case study on Chinese Australians. Health & social care in the community. 2018;26(2):E225-E32. | Interview | 4 | N.R. | N.R. | Migrants | T2DM |
| Choi TST, Walker KZ, Ralston RA, Palermo C. Diabetes education needs of Chinese Australians: A qualitative study. Health Education Journal. 2015;74(2):197-208. | Interview | 8 | 3(37.5)/5(62.5) | 42-80 | Migrants | T2DM |
| Clark L, Vincent D, Zimmer L, Sanchez J. Cultural values and political economic contexts of diabetes among low-income Mexican Americans. Journal of Transcultural Nursing. 2009;20(4):382-94. | Focus Group | 20 | 5(25)/15(75) | 53 | Ethnic Group | T2DM |
| Claydon A, Campbell-Richards D, Hill M. Living with diabetes: A qualitative review of minority ethnic groups in a deprived London borough. Journal of Diabetes Nursing. 2013;17(3):95-100. | Review | n.a. | n.a. | n.a. | n.a. | n.a. |
| Cross-Bardell L, George T, Bhoday M, Tuomainen H, Qureshi N, Kai J. Perspectives on enhancing physical activity and diet for health promotion among at-risk urban UK South Asian communities: a qualitative study. BMJ Open. 2015; 5(2):e007317. doi: 10.1136/bmjopen-2014-007317 PMID: 25724983 | Interview | 34 | 41 | 11(32.4)/23(67.6) | Migrants | T2DM |
| Cusi K, Ocampo GL. Unmet needs in Hispanic/Latino patients with type 2 diabetes mellitus. The American journal of medicine. 2011;124(10 Suppl):S2-9. | Review | n.a. | n.a. | n.a. | n.a. | n.a. |
| Davachi S, Ferrari I. Homelessness and diabetes: reducing disparities in diabetes care through innovations and partnerships. Canadian journal of diabetes. 2012;36(2):75-82. | Diabetes prevention courses | 524 | 388(74)/126(24) | N.R. | Homeless people | T2DM |
| Dayyani I, Terkildsen Maindal H, Rowlands G, Lou S. A qualitative study about the experiences of ethnic minority pregnant women with gestational diabetes. Scand J Caring Sci. 2019. | Interview | 11 | 0(0)/11(100) | 30.9 | Migrants | GDM |
| Detz A, Mangione CM, Nunez de Jaimes F, Noguera C, Morales LS, Tseng CH, et al. Language concordance, interpersonal care, and diabetes self-care in rural Latino patients. Journal of general internal medicine. 2014;29(12):1650-6. | Telephone-based Questionnaire | 250 | 105(42)/145(58) | 54.4 | Migrants | T2DM |
| Early KB, Shultz JA, Corbett C. Assessing Diabetes Dietary Goals and Self-Management Based on In-Depth Interviews With Latino and Caucasian Clients With Type 2 Diabetes. Journal of Transcultural Nursing. 2009;20(4):371-81. | Interview | 10 | 1(10)/9(90) | 40-49 2(20) 50-59 5(50) 60-69 3(30) | Ethnic Group | T2DM |
| Fitzner K, Dietz DA, Moy E. How innovative treatment models and data use are improving diabetes care among older African American adults. Population health management. 2011;14(3):143-55. | Systematic Review | n.a. | n.a. | n.a. | n.a. | n.a. |
| Fritz HA. Learning to do Better: The Transactional Model of Diabetes Self-Management Integration. Qualitative health research. 2015;25(7):875-86.  Fritz HA. Challenges to developing diabetes self-management skills in a low-income sample in North Carolina, USA. Health & social care in the community. 2017;25(1):26-34. | Interview | 10 | 0(0)/10(100) | 45-63 | Low socio-economic status | T2DM |
| Fukuoka Y, Vittinghoff E, Hooper J. A weight loss intervention using a commercial mobile application in Latino Americans-Adelgaza Trial. Translational Behavioral Medicine. 2018;8(5):714-23. | Intervention | 54 | 17(31)/37(69) | 45.3 | Migrants | T2DM |
| Gele AA, Torheim LE, Pettersen KS, Kumar B. Beyond Culture and Language: Access to Diabetes Preventive Health Services among Somali Women in Norway. Journal of diabetes research. 2015;2015:549795. | Interview | 30 | 0(0)/30(100) | N.R. | Migrants | T2DM |
| Grace C, Begum R, Subhani S, Kopelman P, Greenhalgh T. Prevention of type 2 diabetes in British Bangladeshis: qualitative study of community, religious, and professional perspectives. BMJ (Clinical research ed). 2008;337:a1931. | Focus Group | 80 | 37(46)/43(54) | 35 | Migrants | T2DM |
| Guell C. Diabetes management as a Turkish family affair: Chronic illness as a social experience. Annals of Human Biology. 2011;38(4):438-44. | Interview | 7 | N.R. | N.R. | Migrants | T2DM |
| Handley MA, Harleman E, Gonzalez-Mendez E, Stotland NE, Althavale P, Fisher L, et al. Applying the COM-B model to creation of an IT-enabled health coaching and resource linkage program for low-income Latina moms with recent gestational diabetes: the STAR MAMA program. Implementation science : IS. 2016;11(1):73. | Focus Group | 22 | 0(0)/22(100) | 31.5 | Migrants | T2DM/GDM |
| Hempler NF, Nicic S, Ewers B, Willaing I. Dietary education must fit into everyday life: A qualitative study of people with a pakistani background and type 2 diabetes. Patient preference and adherence. 2015;9:347-54. | Interview | 12 | 6(50)/6(50) | 34-70 | Migrants | T2DM |
| Ho EY, Chesla CA, Chun KM. Health communication with Chinese Americans about type 2 diabetes. The Diabetes educator. 2012;38(1):67-76. | Review | n.a. | n.a. | n.a. | n.a. | n.a. |
| Ho EY, Tran H, Chesla CA. Assessing the cultural in culturally sensitive printed patient-education materials for Chinese Americans with type 2 diabetes. Health communication. 2015;30(1):39-49. | Review | n.a. | n.a. | n.a. | n.a. | n.a. |
| Hosler AS, Solanki MN, Savadatti S. Assessing Needs and Feasibility of Diabetes Self-management Coaching at Faith-Based Organizations for Indo-Guyanese Immigrants. The Diabetes educator. 2015;41(3):320-7. | Interview | 6 | 4(67)/2(33) | 57.2 | Migrants | T2DM |
| Hu J, Amirehsani K, Wallace DC, Letvak S. Perceptions of Barriers in Managing Diabetes Perspectives of Hispanic Immigrant Patients and Family Members. Diabetes Educator. 2013;39(4):494-503. | Focus Group | 36 | N.R. | 50 | Migrants | T2DM |
| Hu SH, Fu MR, Liu S, Lin YK, Chang WY. Physical Activity Among Chinese American Immigrants with Prediabetes or Type 2 Diabetes. American Journal of Nursing. 2018;118(2):24-32. | Questionnaire und Interview | 100 | 53(53)/47(47) | 63 | Migrants | T2DM |
| Hyman I, Shakya Y, Jembere N, Gucciardi E, Vissandjee B. Provider- and patient-related determinants of diabetes self-management among recent immigrants: Implications for systemic change. Canadian family physician Medecin de famille canadien. 2017;63(2):e137-e44. | Questionnaire | 130 | 59(45)/71(55) | 51.2 | Migrants | T2DM |
| Ingram M The Amadora Project: identifing factors related to the promotion of physical activity among Mexican Americans with diabetes. Am J Health Promot. 2009;23(6):396-402 | Focus Group | 20 | 3(15)/17(85) | 61 | Migrants | T2DM |
| Islam N, Tandon D, Mukherji R, Tanner M, Ghosh K, Alam G, et al. Understanding the Barriers to and  Facilitators of Diabetes Control and Prevention in the New York City Bangladeshi Community: A Mixed-  Methods Approach. Research and Practice. 2012; 102(3):486–490. | Focus Group | 47 | N.R. | N.R. | Migrants | T2DM |
| Jaber LA, et al. Feasibility of group lifestyle intervention for diabetes prevention in Arab Americans. Diabetes Res Clin Pract. 2011;91(3):307–15.  Pinelli, N. R., Brown, M. B., Herman, W. H. and Jaber, L. A. (2011) Family support is associated with success in achieving weight loss in a group lifestyle intervention for diabetes prevention in Arab Americans. Ethnicity & Disease, 21, 480–484. | Intervention | 71 | 27(38)/44(62) | 47 | Migrants | T2DM |
| Jansà M, Diaz M, Franch J, Vidal M, Gomis R. Anthropologic study of immigrant patients with T2DM from Morocco to Spain: practical implications for therapeutic patient education. European Diabetes Nursing. 2010;7(1):24-8. | Interview | 40 | 11(27)/29(73) | 50 | Migrants | T2DM |
| Jiwrajka M, Mahmoud A, Uppal M. A Rohingya refugee's journey in Australia and the barriers to accessing healthcare. BMJ case reports. 2017;2017. | Case Report | 1 | 0(0)/1(100) | 38 | Migrants | T2DM |
| Jones V, Crowe M. How people from ethnic minorities describe their experiences of managing type-2 diabetes mellitus: A qualitative meta-synthesis. International journal of nursing studies. 2017;76:78-91. | Qualitative Meta-Synthese | n.a. | n.a. | n.a. | n.a. | n.a. |
| Keene DE, Guo M, Murillo S. “That wasn't really a place to worry about diabetes”: Housing access and diabetes self-management among low-income adults. Social Science and Medicine. 2018;197:71-7. | Interview | 40 | 21(52.5)/19(47.5) | 51 | Low income and partially homeless | T2DM |
| Khanam S, Costarelli V. Attitudes towards health and exercise of overweight women. Journal of the Royal Society for the Promotion of Health. 2008;128(1):26-30. | Questionnaire und Interview | 25 | 0(0)/25(100) | 30-60 | Migrants | T2DM |
| Khunti K, Hanif W, Karet B, Mughal S, Patel K. A practical approach to managing type 2 diabetes in the UK south Asian population. Diabetes & Primary Care. 2011;13(1):47-55. | Review | n.a. | n.a. | n.a. | n.a. | n.a. |
| Kindarara DM, McEwen MM, Crist JD, Loescher LJ. Health-Illness Transition Experiences With Type 2 Diabetes Self-management of Sub-Saharan African Immigrants in the United States. Diabetes Educator. 2017;43(5):506-18. | Interview | 10 | 5(50)/5(50) | 60.3 | Migrants | T2DM |
| Kofahl C, von dem Knesebeck O, Hollmann J, Mnich E. [Diabetes-specific health literacy: what do Turkish immigrants with diabetes mellitus 2 know about their disease?]. Gesundheitswesen (Bundesverband der Arzte des Offentlichen Gesundheitsdienstes (Germany)). 2013;75(12):803-11. | Interview | 294 | 138(47)/156(53) | 59 | Migrants | T2DM |
| Kohinor MJE, Stronks K, Haafkens JA. Factors affecting the disclosure of diabetes by ethnic minority patients: a qualitative study among Surinamese in the Netherlands. BMC public health. 2011;11. | Interview | 32 | 12(38)/20(62) | 55 | Migrants | T2DM |
| Lachance L, Kelly RP, Wilkin M, Burke J, Waddell S. Community-Based Efforts to Prevent and Manage Diabetes in Women Living in Vulnerable Communities. Journal of community health. 2018;43(3):508-17. | Intervention | 161 | 0(0)/161(100) | 59.5 | Ethnic Group | T2DM |
| Lee LT, Willig AL, Agne AA, Locher JL, Cherrington AL. Challenges to Healthy Eating Practices: A Qualitative Study of Non-Hispanic Black Men Living With Diabetes. The Diabetes educator. 2016;42(3):325-35. | Focus Group | 34 | 34(100)/0(0) | 53.8 | Ethnic Group | T2DM |
| Lehrer HM, Dubois SK, Brown SA, Steinhardt MA. Resilience-based Diabetes Self-management Education: Perspectives From African American Participants, Community Leaders, and Healthcare Providers. The Diabetes educator. 2017;43(4):367-77. | Focus Group | 16 | N.R. | N.R. | Ethnic Group | T2DM |
| Lopez-Class M, Jurkowski J. The limits of self-management: community and health care system barriers among Latinos with diabetes. J Hum Behav Soc Environ. 2010;20(6):808-826. | Review | n.a. | n.a. | n.a. | n.a. | n.a. |
| Lucas A, Murray E, Kinra S. Heath beliefs of UK South Asians related to lifestyle diseases: a review of qualitative literature. Journal of obesity. 2013;2013:827674. | Review | n.a. | n.a. | n.a. | n.a. | n.a. |
| Ludwig AF, Cox P, Ellahi B. Social and cultural construction of obesity among Pakistani Muslim women in North West England. Public health nutrition. 2011;14(10):1842-50. | Focus Group and Interview | 55 | 0(0)/55(100) | 23-80 | Migrants | T2DM |
| Madden MH, Tomsik P, Terchek J, Navracruz L, Reichsman A, Clark TC, et al. Keys to successful diabetes self-management for uninsured patients: social support, observational learning, and turning points: a safety net providers' strategic alliance study. Journal of the National Medical Association. 2011;103(3):257-64. | Interview | 26 | 9(34.6)/17(65.4) | N.R. | unemployed people | T2DM |
| Maglalang DD, Yoo GJ, Ursua RA, Villanueva C, Chesla CA, Bender MS. "I DON'T HAVE TO EXPLAIN, PEOPLE UNDERSTAND": ACCEPTABILITY AND CULTURAL RELEVANCE OF A MOBILE HEALTH LIFESTYLE INTERVENTION FOR FILIPINOS WITH TYPE 2 DIABETES. Ethnicity & disease. 2017;27(2):143-54. | Interview | 45 | 17(38)/28(62) | 57.6 | Migrants | T2DM |
| Maine A, Brown MJ, Dickson A, Truesdale M. Pilot feasibility study of the Walking Away from Diabetes programme for adults with intellectual disabilities in two further education colleges: Process evaluation findings. Journal of Applied Research in Intellectual Disabilities. 2019;32(5):1034-46. | Focus Group | 48 | 30(63)/18(37) | 20.9 | Disabled people | T2DM |
| Majeed-Ariss R, Jackson C, Knapp P, Cheater FM. British-Pakistani women's perspectives of diabetes self-management: the role of identity. Journal of clinical nursing. 2015;24(17-18):2571-80. | Interview | 15 | 0(0)/15(100) | 53.2 | Migrants | T2DM |
| Majeed-Ariss R, Jackson C, Knapp P, Cheater FM. A systematic review of research into black and ethnic minority patients' views on self-management of type 2 diabetes. Health Expectations. 2015;18(5):625-42. | Systematic Review | n.a. | n.a. | n.a. | n.a. | n.a. |
| Marcy TR, Britton ML, Harrison D. Identification of barriers to appropriate dietary behavior in low-income patients with type 2 diabetes mellitus. Diabetes Therapy. 2011;2(1):9-19. | Questionnaire | 98 | 20(20.4)/78(79.6) | 51.98 | Low socio-economic status | T2DM |
| Marquez DX, Bustamante EE, Bock BC, Markenson G, Tovar A, Chasan-Taber L. Perspectives of Latina and non-Latina white women on barriers and facilitators to exercise in pregnancy. Women & health. 2009;49(6):505-21. | Focus Group | 13 | 0(0)/13(100) | 25.1 | Migrants | GDM |
| Martinez J, Powell J, Agne A, Scarinci I, Cherrington A. A focus group study of Mexican immigrant men's perceptions of weight and lifestyle. Public health nursing (Boston, Mass). 2012;29(6):490-8. | Focus Group and Questionnaire | 16 | 16(100)/0(0) | 41 | Migrants | T2DM |
| McCloskey, D., & Flenniken, M. A. (2010). Overcoming cultural barriers to diabetes control: A qualitative study of Southwestern New Mexico Hispanics. Journal of Cultural Diversity, 17(3), 110-115. | Review | n.a. | n.a. | n.a. | n.a. | n.a. |
| McEwen MM, Murdaugh C. Partnering With Families to Refine and Expand a Diabetes Intervention for Mexican Americans. The Diabetes educator. 2014;40(4):488-95. | Focus Group | 12 | 6(50)/6(50) | 58 | Migrants | T2DM |
| Mian SI, Brauer PM. Dietary education tools for South Asians with diabetes. Canadian journal of dietetic practice and research : a publication of Dietitians of Canada = Revue canadienne de la pratique et de la recherche en dietetique : une publication des Dietetistes du Canada. 2009;70(1):28-35. | Focus Group | 53 | 29(54.7)/24(45.3) | 25-29 2 30-39 14 40-49 11 50-59 13 60-69 10 70-79 3 | Migrants | T2DM |
| Miller ST, Marolen K. Physical activity-related experiences, counseling expectations, personal responsibility, and altruism among urban African American women with type 2 diabetes. The Diabetes educator. 2012;38(2):229-35. | Focus Group | 11 | 0(0)/11(100) | 63.6% are 41-50 years old | Ethnic Group | T2DM |
| Miller ST, Marolen KN, Beech BM. Perceptions of physical activity and motivational interviewing among rural African-American women with type 2 diabetes. Women's health issues : official publication of the Jacobs Institute of Women's Health. 2010;20(1):43-9. | Focus Group | 31 | 0(0)/31(100) | 50% are 41-50 years old | Ethnic Group | T2DM |
| Mitchell-Brown F, Nemeth L, Cartmell K, Newman S, Goto K. A Study of Hmong Immigrants’ Experience With Diabetes Education: A Community-Engaged Qualitative Study. Journal of Transcultural Nursing. 2017;28(6):540-9. | Focus Group | 16 | 7(43.8)/9(56.2) | 55.1 | Migrants | T2DM |
| Moise RK, Conserve DF, Elewonibi B, Francis LA, BeLue R. Diabetes Knowledge, Management, and Prevention Among Haitian Immigrants in Philadelphia. The Diabetes educator. 2017;43(4):341-7. | Focus Group | 10 | 3(30)/7(70) | 65 | Migrants | T2DM |
| Mora N, Golden SH. Understanding Cultural Influences on Dietary Habits in Asian, Middle Eastern, and Latino Patients with Type 2 Diabetes: A Review of Current Literature and Future Directions. Current diabetes reports. 2017;17(12):126. | Review | n.a. | n.a. | n.a. | n.a. | n.a. |
| Murrock CJ, Taylor E, Marino D. Dietary challenges of managing type 2 diabetes in African-American women. Women & health. 2013;53(2):173-84. | Focus Group | 24 | 0(0)/24(100) | 47.7 | Ethnic Group | T2DM |
| Nam S, Song H-J, Park S-Y, Song Y. Challenges of diabetes management in immigrant Korean Americans. The Diabetes educator. 2013;39(2):213-21. | Focus Group | 23 | 14(60.9)/9(39.1) | 58.5 | Migrants | T2DM |
| Newlin K, Dyess SM, Allard E, Chase S, Melkus GD. A methodological review of faith-based health promotion literature: advancing the science to expand delivery of diabetes education to Black Americans. Journal of religion and health. 2012;51(4):1075-97. | Review | n.a. | n.a. | n.a. | n.a. | n.a. |
| Njeru JW, Patten CA, Hanza MM, Brockman TA, Ridgeway JL, Weis JA, et al. Stories for change: development of a diabetes digital storytelling intervention for refugees and immigrants to minnesota using qualitative methods. BMC public health. 2015;15:1311. | Focus Group | 37 | 21(56.8)/16(43.2) | 55.8 | Migrants | T2DM |
| O'Brien MJ, Shuman SJ, Barrios DM, Alos VA, Whitaker RC. A qualitative study of acculturation and diabetes risk among urban immigrant Latinas: implications for diabetes prevention efforts. The Diabetes educator. 2014;40(5):616-25. | Focus Group | 26 | 0(0)/26(100) | N.R. | Migrants | T2DM |
| Onwudiwe NC, Mullins CD, Winston RA, Shaya FT, Pradel FG, Laird A, et al. Barriers to self-management of diabetes: a qualitative study among lowincome minority diabetics. Ethn Dis 2011;21(1):27-32. | Focus Group | 31 | N.R. | 74 | Ethnic Group | T2DM |
| Oza-Frank R, Conrey E, Bouchard J, Shellhaas C, Weber MB. Healthcare Experiences of Low-Income Women with Prior Gestational Diabetes. Maternal and child health journal. 2018 | Focus Group | 12 Groups with a mean of 7.2 participants/group | All female | N.R. | Migrants/Ethnic Group | T2DM/GDM |
| Pan W, Ge SQ, Xu Y, Toobert D. Cross-Validating a Structural Model of Factors Influencing Diabetes Self-Management in Chinese Americans with Type 2 Diabetes. Journal of Transcultural Nursing. 2019;30(2):163-72. | Questionnaires | 209 | 82(39)/127(61) | 68.3 | Migrants | T2DM |
| Park C, Nam S, Whittemore R. Incorporating Cultural Perspectives into Diabetes Self-Management Programs for East Asian Immigrants: A Mixed-Study Review. Journal of immigrant and minority health. 2016;18(2):454-67. | Systematic Review | n.a. | n.a. | n.a. | n.a. | n.a. |
| Patel, M., Phillips-Caesar, E. and Boutin-Foster, C. (2011) Barriers to lifestyle behavioral change in migrant South Asian populations. Journal of Immigrant and Minority Health, 14, 774–785. | Review | n.a. | n.a. | n.a. | n.a. | n.a. |
| Patel V, Iliffe S. An exploratory study into the health beliefs and behaviours of British Indians with type II diabetes. Primary Health Care Research and Development. 2017;18(1):97-103. | Interview | 10 | 8(80)/2(20) | 61 | Ethnic Group | T2DM |
| Patel N, Ferrer HB, Tyrer F, Wray P, Farooqi A, Davies MJ, et al. Barriers and Facilitators to Healthy Lifestyle Changes in Minority Ethnic Populations in the UK: a Narrative Review. Journal of racial and ethnic health disparities. 2017;4(6):1107-19. | Review | n.a. | n.a. | n.a. | n.a. | n.a. |
| Pilkington FB, Daiski I, Bryant T, Dinca-Panaitescu M, Dinca-Panaitescu S, Raphael D. The experience of living with diabetes for low-income Canadians. Canadian journal of diabetes. 2010;34(2):119-26. | Interview | 58 | 24(41.4)/34(58.6) | 57 | Low socio-economic status | T2DM |
| Plasencia J, Hoerr S, Carolan M, Weatherspoon L. Acculturation and Self-Management Perceptions Among Mexican American Adults With Type 2 Diabetes. Family & community health. 2017;40(2):121-31. | Interview | 24 | 11(45.8)/13(54.2) | N.R. | Migrants | T2DM |
| Purcell N, Cutchen L. Diabetes Self-Management Education for African Americans: Using the PEN-3 Model to Assess Needs. American Journal of Health Education. 2013;44(4):203-12. | Focus Group | 16 | 8(50)/8(50) | 52.7 | Ethnic Group | T2DM |
| Pyatak EA, Florindez D, Peters AL, Weigensberg MJ. "We Are All Gonna Get Diabetic These Days" The Impact of a Living Legacy of Type 2 Diabetes on Hispanic Young Adults' Diabetes Care. Diabetes Educator. 2014;40(5):648-58. | Interview | 8 | 3(37.5)/5(62.5) | 20.6 | Migrants | T2DM |
| Rahim-Williams B. Beliefs, behaviors, and modifications of type 2 diabetes self-management among African American women. Journal of the National Medical Association. 2011;103(3):203-15. | Questionnaire and Interview | 24 | 0(0)/24(100) | 64 | Ethnic Group | T2DM |
| Ramal E, Petersen AB, Ingram KM, Champlin AM. Factors that influence diabetes self-management in Hispanics living in low socioeconomic neighborhoods in San Bernardino, California. Journal of immigrant and minority health. 2012;14(6):1090-6. | Focus Group | 27 | 6(22.2)/21(77.8) | N.R. | Migrants | T2DM |
| Raphael D, Daiski I, Pilkington B, Bryant T, Dinca-Panaitescu M, Dinca-Panaitescu S. A toxic combination of poor social policies and programmes, unfair economic arrangements and bad politics: the experiences of poor Canadians with Type 2 diabetes. Critical Public Health. 2012;22(2):127-45. | Interview | 60 | 24(41.4)/34(58.6) | 30-39 3 40-49 9 50-59 23 60-69 11 70-79 11 >80 2 | Low socio-economic status | T2DM |
| Rendle KAS, May SG, Uy V, Tietbohl CK, Mangione CM, Frosch DL. Persistent barriers and strategic practices: Why (asking about) the everyday matters in diabetes care. The Diabetes educator. 2013;39(4):560-7. | Interview | 20 | 11(55)/9(45) | 56 | Low socio-economic status | T2DM |
| Rhoads-Baeza ME, Reis J. An exploratory mixed method assessment of low income, pregnant Hispanic women's understanding of gestational diabetes and dietary change. Health Education Journal. 2012;71(1):80-9. | Interview | 94 | 0(0)/94(100) | 18-31 | Migrants | GDM |
| Richardson BS, Willig AL, Agne AA, Cherrington AL. Diabetes connect: African American women’s perceptions of the community health worker model for diabetes care. Journal of Community Health: The Publication for Health Promotion and Disease Prevention. 2015;40(5):905-11. | Focus Group | 25 | 0(0)/25(100) | N.R. | Ethnic Group | T2DM |
| Rosal MC, Borg A, Bodenlos JS, Tellez T, Ockene IS. Awareness of diabetes risk factors and prevention strategies among a sample of low-income Latinos with no known diagnosis of diabetes. The Diabetes educator. 2011;37(1):47-55. | Focus Group | 41 | 6(15)/35(85) | 44.3 | Migrants | T2DM |
| Ruddock JS, Poindexter M, Gary-Webb TL, Walker EA, Davis NJ. Innovative strategies to improve diabetes outcomes in disadvantaged populations. Diabetic medicine: a journal of the British Diabetic Association. 2016;33(6):723-33. | Review | n.a. | n.a. | n.a. | n.a. | n.a. |
| Saunders T. Type 2 Diabetes Self-Management Barriers in Older Adults An Integrative Review of the Qualitative Literature. Journal of Gerontological Nursing. 2019;45(3):43-54. | Review | n.a | n.a | n.a. | n.a. | n.a. |
| Shacter HE, Shea JA, Akhabue E, Sablani N, Long JA. A qualitative evaluation of racial disparities in glucose control. Ethnicity & disease. 2009;19(2):121-7. | Group discussion | 33 | N.R. | N.R. | Older people | T2DM |
| Shultz JA, Corbett CF, Allen CB. Slavic Women's Understanding of Diabetes Dietary Self-Management and Reported Dietary Behaviors. Journal of immigrant and minority health. 2009;11(5):400-5. | Interview | 10 | 0(0)/10(100) | N.R. | Migrants/Low socio-economic status | T2DM |
| Singh H, Cinnirella M, Bradley C. Support systems for and barriers to diabetes management in South Asians and Whites in the UK: Qualitative study of patients' perspectives. BMJ open. 2012;2(6). | Interview | 12 | 6(50)/6(50) | N.R. | Migrants | T2DM |
| Smith-Miller CA, Berry DC, Miller CT. Diabetes affects everything: Type 2 diabetes self-management among Spanish-speaking hispanic immigrants. Research in nursing & health. 2017;40(6):541-54. | Interview | 30 | 11(37)/19(63) | 45 | Migrants | T2DM |
| Sohal PS. Prevention and management of diabetes in South Asians. Canadian journal of diabetes. 2008;32(3):206-10. | Review | n.a. | n.a. | n.a. | n.a. | n.a. |
| Sohal T, Sohal P, King-Shier KM, Khan NA. Barriers and facilitators  for type-2 diabetes management in South Asians: a systematic  review. PLoS One. 2015;10(9):e0136202. https://doi.org/10.1371/  journal.pone.0136202. | Review | n.a. | n.a. | n.a. | n.a. | n.a. |
| Song Y, Song HJ, Han HR, Park SY, Nam S, Kim MT. Unmet Needs for Social Support and Effects on Diabetes Self-care Activities in Korean Americans With Type 2 Diabetes. Diabetes Educator. 2012;38(1):77-85. | Questionnaire | 83 | 48(57.8)/35(42.2) | 56.5 | Ethnic Group | T2DM |
| Sullivan LV, Hicks P, Salazar G, Robinson CK. Patient beliefs and sense of control among Spanish-speaking patients with diabetes in northeast Colorado. Journal of immigrant and minority health. 2010;12(3):384-9. | Focus Group | 35 | 10(29)/25(71) | 46 | Migrants | T2DM |
| Tang J, Foster K, Pumarino J, Ackermann R, Peaceman A, Cameron K. Perspectives on Prevention of Type 2 Diabetes After Gestational Diabetes: A Qualitative Study of Hispanic, African-American and White Women. Maternal & Child Health Journal. 2015;19(7):1526-34. | Interview | 23 | 0(0)/23(100) | 33.1 | Migrants/Ethnic Group | T2DM |
| Wang Y, Chuang L, Bateman WB. Focus group study assessing self-management skills of Chinese Americans with type 2 diabetes mellitus. Journal of immigrant and minority health. 2012;14(5):869-74. | Focus Group | 24 | 16(66.7)/6(25) | N.R. | Migrants | T2DM |
| Weiler DM, Crist JD. Diabetes self-management in a Latino social environment. The Diabetes educator. 2009;35(2):285-92. | Interview | 10 | 4(40)/6(60) | 46-65 | Migrants | T2DM |
| Yee LM, McGuire JM, Taylor SM, Niznik CM, Simon MA. Social and environmental barriers to nutrition therapy for diabetes management among underserved pregnant women: A qualitative analysis. Journal of nutrition education and behavior. 2016;48(3):170-80.  Yee LM, McGuire JM, Taylor SM, Niznik CM, Simon MA. Factors promoting diabetes self-care among low-income, minority pregnant women. Journal of Perinatology. 2016;36(1):13-8. | Interview | 10 | 0(0)/10(100) | 28.5 | Low socio-economic status | T2DM/GDM |
| Yeoh S, Furler J. Perceptions of Health and Diabetes in a Melbourne South Sudanese Community. Journal of Immigrant & Minority Health. 2011;13(5):914-8. | Focus Group | 25 | 6(24)/19(76) | N.R. | Migrants | T2DM |
| Yilmaz-Aslan Y, Brzoska P, Bluhm M, Aslan A, Razum O. Illness perceptions in Turkish migrants with diabetes: a qualitative study. Chronic illness. 2014;10(2):107-21. | Interview | 24 | 11(45.9)/13(54.1) | 60.3 | Migrants | T2DM |
| Zeng B, Sun WJ, Gary RA, Li CW, Liu TT. Towards a Conceptual Model of Diabetes Self-Management among Chinese Immigrants in the United States. International journal of environmental research and public health. 2014;11(7):6727-42. | Review | n.a. | n.a. | n.a. | n.a. | n.a. |

GDM: gestational diabetes; n.a.: not applicable; N.R.: not reported; T2DM: type 2 diabetes mellitus
